# Bindings and RESTlets: A Novel Set of CoAP-Based Application Enablers to Build IoT Applications

**DOI:** 10.3390/s16081217

**Published:** 2016-08-02

**Authors:** Girum Ketema Teklemariam, Floris Van Den Abeele, Ingrid Moerman, Piet Demeester, Jeroen Hoebeke

**Affiliations:** 1 Department of Information Technology (INTEC), Ghent University—iMinds, Technologiepark-Zwijnaarde 15, 9052 Ghent, Belgium; floris.vandenabeele@intec.ugent.be (F.V.D.A.); ingrid.moerman@intec.ugent.be (I.M.); piet.demeester@intec.ugent.be (P.D.); jeroen.hoebeke@intec.ugent.be (J.H.); 2 Information Communication Technology Development Directorate, Jimma University, P.O. Box 378, Jimma, Ethiopia

**Keywords:** Internet of Things, CoAP, sensor/actuator binding, RESTlets, IoT application, in-network processing, resource observation

## Abstract

Sensors and actuators are becoming important components of Internet of Things (IoT) applications. Today, several approaches exist to facilitate communication of sensors and actuators in IoT applications. Most communications go through often proprietary gateways requiring availability of the gateway for each and every interaction between sensors and actuators. Sometimes, the gateway does some processing of the sensor data before triggering actuators. Other approaches put this processing logic further in the cloud. These approaches introduce significant latencies and increased number of packets. In this paper, we introduce a CoAP-based mechanism for direct binding of sensors and actuators. This flexible binding solution is utilized further to build IoT applications through RESTlets. RESTlets are defined to accept inputs and produce outputs after performing some processing tasks. Sensors and actuators could be associated with RESTlets (which can be hosted on any device) through the flexible binding mechanism we introduced. This approach facilitates decentralized IoT application development by placing all or part of the processing logic in Low power and Lossy Networks (LLNs). We run several tests to compare the performance of our solution with existing solutions and found out that our solution reduces communication delay and number of packets in the LLN.

## 1. Introduction

In the information age that we are living in, we have witnessed remarkable advances in electromechanical technologies, miniaturization and wireless communication. It is not uncommon to see tiny and very powerful electromechanical devices that can be integrated in our environment by embedding them in everyday objects around us and are capable of doing things that were unimaginable a few decades ago. Sensor and actuator nodes are best examples of such tiny devices that have become parts of our daily life for quite some time. Sensor nodes are devices that capture events in the physical world and transfer them into the virtual world as raw data so that they can be processed and acted upon. Actuators, on the other hand, alter the real world based on data obtained from the physical world or cyber world, periodically or spontaneously. It is very common to see sensors and actuators working together. Motion activated doors, air conditioners, environmental monitoring systems, and voice activated security systems are some examples which involve both sensors and actuators. The communication arena has also seen remarkable changes in the past couple of decades. Billions of devices are being interconnected with each other around the globe. 

These advancements of both communication and electromechanical technologies have led to new possibilities for those tiny devices. Sensors can be connected to other actors such as services that process the raw data and subsequently interact with actuators without regard for physical proximity. In the beginning, these solutions were characterized by proprietary solutions implemented by different vendors. Such solutions either involve proprietary intermediary devices between the sensors and actuators for data processing or require static configuration to be done to allow direct interactions. The former approach forces users to stick to one vendor due to lack of interoperability. In addition, when using multiple vendors, it results in several vertical silos for different applications. The latter approach also has its own limitation such as limited reuse, complexity in programming, and limited integration with applications.

Since the last decade, diverse initiatives have been launched by various organizations in order to make these devices an integral part of the Internet. The Internet Engineering Task Force (IETF) has been the pioneer by establishing a number of focused working groups that address various aspects of the integration. Due to the constraints of the tiny devices such as limited processing, storage, transmission and reception capacity and limited power availability, existing Internet networking protocols were not directly suitable. Therefore, various solutions have been proposed at the different layers of the Open Systems Interconnection (OSI) model. For instance, IPv6 packets are too big to be processed, transmitted, and received in an energy efficient way by the constrained devices. To this end, the 6LoWPAN adaptation layer has been developed for compression/decompression and transmission of IPv6 packets so that they can be transported over constrained networks [1]. Another issue that needs to be addressed is routing. The routing protocols used in today’s Internet are all designed to find optimal paths to destinations by considering the routers to be powerful enough to store and process large routing tables. This makes them not suitable for constrained multi-hop networks. Moreover, the metrics that should be considered in constrained networks are considerably different from those in non-constrained networks. As a result, a new routing protocol named Routing Protocol for LLN (RPL) has been proposed [2]. 

Finally, also the application layer needs some adaptations to enable efficient integration. As described in [3], web services are ideal for machine to machine communication. Unfortunately, the existing protocols, such as HTTP, used for web services are too heavy and verbose for constrained nodes. For this reason, a lightweight RESTful application protocol, Constrained Application Protocol (CoAP), has been proposed [4]. CoAP provides similar functionality for constrained networks as the one HTTP provides for conventional networks. CoAP uses PUT, POST, GET, and DELETE methods to communicate, update or remove resources hosted by the sensor/actuator nodes. 

To maximize the benefit of web services using CoAP, a number of extensions are proposed. The most relevant extension to our work is resource observation [5]. One of the application areas of Sensor/Actuator Networks is monitoring of different environmental phenomena. In such systems, sensors gather information and send it to a monitoring station so that the appropriate action can be taken. The communication between the sensor and the monitoring station can be done through frequent polling, but results in several unnecessary request/response pairs if the values do not change frequently. By adding an observe mechanism, sensors can inform interested parties about any state changes of resources they want to observe. Communication can be optimized further by sending notification criteria while registering for observation. This way only changes that have significant importance to the observer are communicated. Details of this method, called Conditional Observation, can be found in [6]. All in all, CoAP and its extensions enable the interaction with constrained devices in a RESTful way over IP networks. This interaction can be utilized further to build IoT applications that make use of sensor data or steer actuators. This can be achieved by interacting with constrained devices within browsers [7] or services running in the cloud. 

This paper focuses on novel enablers on top of CoAP to facilitate the creation and configuration of IoT applications with low creation and configuration overhead. The first step in building such IoT applications is to enable direct interactions between constrained devices without the need or continuous presence of an intermediary. This option is not yet available in CoAP and represents one of the contributions of this paper, namely binding sensors and actuators so that they can directly interact with each other, eliminating the need for external devices to continuously coordinate communication between them. We show how the CoAP protocol and the observe option (along with the conditional observe extension) can be used to create such direct interactions, also called *bindings*, between sensors and actuators in a flexible way. The interactions themselves are fully RESTful CoAP-based interactions, allowing anything to be bound to anything. This offers a lot more flexibility than other binding solutions presented so far. On top of that, we propose configurable, connectable and reusable building blocks with CoAP interfaces, called *RESTlets* that perform some processing of data at different levels. The processing can be done inside the constrained network, at the edge of the constrained network or in the Cloud. It acts as an enabler to build IoT applications that consist of modular processing steps, potentially distributed along the path between the sensors and the Cloud. The RESTlets can accept inputs and produce outputs after performing basic processing and can be configured through control parameters. To link sensors and actuators to RESTlets or to create chains of RESTlets, we build upon the binding concept. This contribution shows that creation of (part of) an IoT application can be reduced to linking together devices and processing blocks, demonstrating feasibility of the approach. In addition, we investigate the performance in terms of overhead and how this can by optimized by looking at different options such as location of RESTlets, in-network processing, etc.

This paper is organized in seven sections. The underlying protocol, CoAP is discussed in more detail in the next section and related work will be discussed after that. The binding concept will be discussed in Section 4 followed by a discussion of the RESTlet concept. Section 6 shows the implementation and evaluation of the two concepts. The paper ends by discussing main findings and giving concluding remarks.

## 2. Constrained Application Protocol (CoAP) 

Recently, IETF approved the Constrained Application Protocol (CoAP) [4] as an open standard suitable for machine-to-machine communication or IoT interactions. As it implements a subset of the Representational State Transfer (REST) paradigm, it is referred to as the lightweight counterpart of HTTP. CoAP employs the same four methods, namely GET, PUT, POST and DELETE, as HTTP when sending requests from a client to a server. However, unlike HTTP, CoAP uses UDP as transport layer protocol in order to avoid the message overhead and extensive resource requirements of TCP. Reliability is provided through confirmable messages, allowing a client to specify whether a message should be acknowledged or not.

**Figure 1:**
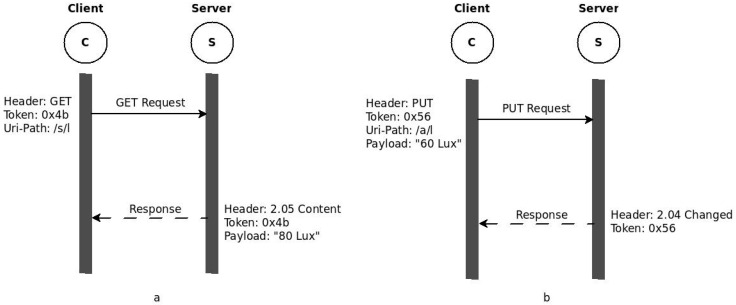
(**a**) CoAP GET Operation; (**b**) CoAP PUT Operation.

CoAP client/server communication takes place in the same way as any REST-based communication. Clients send a request to a specific resource identified by a URI to retrieve the current resource representation or to modify it. The server then replies with the current representation or a status message. For instance, as shown in Figure 1a, if a client would like to receive the current light intensity in a room, it just sends a GET request to the specific resource representing the light intensity. Upon receipt, the sensor responds with the current value. Figure 1a shows this client/server interaction where the resource is represented by /s/l and the current value, which is 80 Lux, is sent back to the client. The responses could be piggybacked or separate. Piggybacked responses contain the data along with the acknowledgement to a confirmable request. However, if the data is not available during the request, for various reasons, the acknowledgement is sent alone and the response follows when the node is ready to send the data. The other methods may not require data to be sent back, but responses that indicate the outcome of message will be returned. Figure 1b shows, a client that sends PUT to a resource, represented by /a/l, on a device such as a Dimmer to change the light intensity to 60 Lux. After adjusting the value, the dimmer responds with a status code, stating that the value is changed.

Responses can also be cached at intermediaries to improve efficiency. This feature is especially important for constrained devices as proxying will allow other devices to respond in place of the constrained node which might be sleeping or to limit traffic in the constrained network.

**Figure 2:**
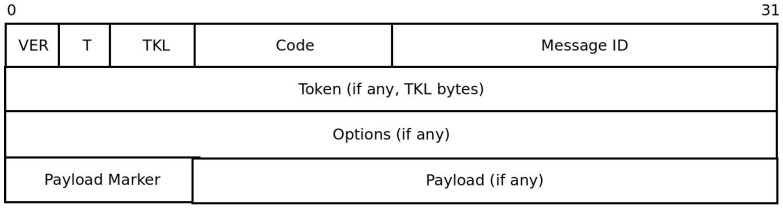
CoAP Header.

CoAP requests and responses use a fixed four bytes header followed by zero or more compact binary options and an optional payload (Figure 2). The VER field (2 bits) in the header indicates CoAP version number and is always set to 1. The T field (2 bits) indicates message type. Message type 0 indicates CON (confirmable) request; and is used if reliability is required. Requests sent as CON must be acknowledged by setting the message type to ACK (value = 2). On the other hand, non-confirmable (NON) messages (Value 1), are used if reliability is not a requirement. Token length (TKL) field is part of the four bytes CoAP header and indicate the length of the token which immediately follows the fixed CoAP header. The Token is used to match requests with responses. Code (8 bits) field is used to indicate request method in requests and response code in responses. Message ID (16 bits) is used to detect duplicates and also match requests with responses. CoAP packets may contain one or more options to specify different aspects of the message such as URI and message format. Options are inserted in packets in ascending order and specified as option delta, length and value. If the message contains payload to be transmitted, an eight bit Payload Marker field is inserted followed by the payload. Refer to the CoAP RFC [4] for detailed explanation of the fields and CoAP operations.

**Figure 3:**
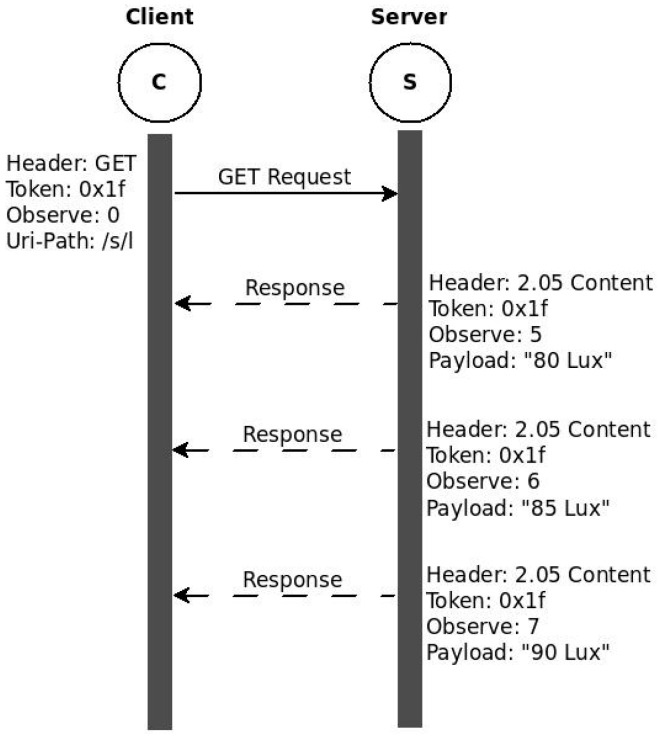
CoAP Observe Operation.

As mentioned above, CoAP is designed following the REST architecture and is optimized for M2M communication in constrained environments. One of the application areas where M2M communication is widely used is resource monitoring. In such applications, clients need to have an up-to-date representation of data from servers. Polling (sending periodic requests to servers) is not optimal in constrained environments since the values may not change as often as the polling request frequency resulting in unnecessary packet transmissions. Introducing a mechanism that triggers transmissions only if changes occur improves the communication significantly. Resource Observation [5] is an interesting extension of the CoAP protocol that introduces such a mechanism. With the observe extension, clients can inform servers about their interest in getting an up-to-date representation of a resource by adding the observe option along their GET request. As a result, the server registers the client as an observer and sends the current representation. After that, the server only sends values, called notifications, when there is change in the resource representation. This method of communication is proposed based on the well-known observer design model. Figure 3 shows this communication model by taking the light sensor example discussed above. Instead of repeatedly requesting for the current representation, the client sends a GET with an observe option. This is called registration at the server. The server responds by including the current representation and registering the client as an observer. Subsequent changes in the resource representation will trigger notifications to be sent back to the client. 

In this communication model, the original request and subsequent notifications are matched using the Token. In frequently changing environments, the representations may arrive out of order at the client. The observe option value is incremented each time to identify the latest value. For a resource to be observed by clients, it must be defined as observable, also indicated by the obs attribute in the resource definition.

A significant performance gain is obtained by using resource observation instead of polling. Nevertheless, not every event change might be significant enough for the client to store it or to trigger any action. Therefore, all those insignificant messages will be dropped after being transmitted over the constrained network. Those unnecessary transmissions can be suppressed by combining resource observation with server side filtering. Conditional Observation [6] provides a mechanism for clients to specify notification criteria during registration. As a result, servers will filter notifications and send only those that meet the notification criteria. Detailed implementation and evaluation of Conditional observation is given in [6]. The core-interfaces draft [8] also allows server side filtering by allowing clients to specify notification criteria in URI-Queries.

## 3. Related Work

### 3.1. Sensor-Actuator Interaction

The traditional approach for applications that require sensor-actuator interaction was to statically associate related devices at the time of deployment. This approach lacks flexibility. One of the earliest works that attempts flexible binding is the ZigBee End Device binding [9]. As stated in the specification, devices with similar End Device Profile and matching cluster ID can be bound. This dynamic binding method places many strict requirements on the devices that will be bound and, as a result, lacks the flexibility of binding any device with any other device that our solution provides. Another notable work that attempts to improve the limitation of ZigBee End Device Binding is given on [10]. This work avoids the requirement of matching cluster ID by matching sensor events with actuator actions. However, this solution is also based on ZigBee and devices that are not compatible with ZigBee cannot be included in the binding process and hence still lacks the flexibility we desire. To achieve maximum flexibility of binding any two devices, working on open standards is preferable. In line with this, the CoRE Interfaces draft [8] specifies how CoAP methods can be used to achieve flexible binding. The mechanism proposed in the draft allows end devices to establish a binding relationship between two resources through discovery mechanisms or through human intervention and then synchronizes the content of the involved resources. This solution has its advantages as it provides a generic solution that can be used in interface descriptions. However, the solution focuses on synchronizing the contents of two resources on different end devices. It is not possible to execute a specific action on the other device. Additional programming logic is still required to send the appropriate trigger to the same or different actuator.

### 3.2. In-Network Processing

Different developers have suggested different IoT application development models. Some prefer WS-* such as SOAP using HTTP while others argue that RESTful approaches are better suited for IoT [11]. A survey conducted among developers [12] concluded that RESTful web services were the preferred choice of most developers. However, even RESTful IoT applications have different development approaches. Traditional applications have been running at the edge of the constrained network or on the gateway. In recent years, many applications are moving into the cloud [13]. Actinium [13] is one such solution. Actinium divides the whole IoT application into Thin Servers that provide hardware functionality through RESTful interfaces and scripted apps, which run in the cloud and implement the IoT application logic. This allows developers to focus on programming their application to run on the cloud without dealing with the constrained environment. This approach is significantly different from our approach which attempts to do as much processing as possible inside the LLN e.g., in order to reduce latency, to limit the amount of data going to the Cloud or to remain operational in the absence of connectivity. 

There are other initiatives, similar to ours, which attempt to keep some or all of the processing logic inside the LLN. Ref. [14] presents a programming abstraction known as *T-Res* which models processing tasks as resources that sit on a constrained device and can be manipulated by CoAP methods. Each T-Res resource stores URIs of the input and output devices as sub-resources. The last output and the compiled processing function (originally written in Python) are also stored as sub-resources. The processing function internally connects the input sources and output destinations by reading data from the input source(s) and sending out new outputs to devices identified by the URLs, if any. The last output is stored to allow concatenation of tasks. T-RES also provides getter and setter functions as programing APIs to be used in processing functions. Even though this system has some similarities to our solution, there are quite many significant differences, the first one being the overall approach. This solution represents processing tasks as resources while we model RESTlets to be independent IoT application building blocks that may run anywhere in the network (inside LLN, on Gateway or in the Cloud). We also store input that may arrive from any device and send stored output to any other device after processing, but take a different approach regarding the way processing is done. In case of T-Res, the processing function is responsible for getting the inputs from the sensors and sending the output, if any. Our solution separates input retrieval and task processing by using flexible binding for the interaction with sensors. Another difference, which is also a significant limitation of this solution, arises from the very architecture of the solution. Every task resource stores URLs of input sources and destination outputs. This means if the application requires doing the same processing task (e.g., Average) on different sets of sensors and/or sends output to different actuators, multiple task resources needs to be defined and the same function will have to be stored in each resource. Our system enables reuse of processing functionalities as long as the processing logic remains the same. 

In [15], virtual sensors and actuators are defined as resources to provide a mechanism to move part of the IoT application processing logic into the LLN. The virtual resources are defined hierarchically as template, instance and configuration resources. Whenever a new virtual resource is created on a device, the template must be posted on the virtual resource directory and the corresponding sub-resources. The input is pulled and calculation and generation of notification is done only when GET is issued by a component of the code in the cloud. Our solution interconnects the devices to talk to each other automatically without the need for external commands. Further, this solution still has its components in the cloud while ours tries to avoid putting the application code in cloud as much as possible. LooCi [16] is another model for IoT applications. It uses an event-based binding model and standardized event types that allow easy component interactions and re-use of components. This approach uses Remote Protocol Call (RPC) for communication.

## 4. Flexible Direct Binding

In many IoT applications, sensors and actuators are deployed to work together. Examples of such applications include temperature sensors and thermostats, a light switch (sensor) and light bulb (actuator) in smart lighting solutions, and motion sensors and automatic door controls. Some old installations use static configurations where sensors are associated with fixed actuator(s) before or during deployment. This solution allows direct interaction between devices but has serious flexibility issues. If we need to change the association made during deployment, we need to reconfigure the devices all over again. Another solution often used in many applications is introducing an, often proprietary, intermediary between the sensor and actuator. In such solutions, the sensor sends the data to the intermediary device and the device sends the trigger to the actuator. This indirect communication involves too many unnecessary transmissions of packets between the three devices. In addition to this, every communication needs to move to the edge of the LLN (or even further into the cloud), resulting in delayed response due to higher latency. Moreover, if the intermediary device is down for any reason, the whole communication between the devices will not take place.

In this section, we introduce the concept of flexible direct bindings, which solves the aforementioned problems by allowing direct interaction of smart objects without losing flexibility. To avoid vendor lock-in and allow devices from different vendors to communicate with each other, the implementation of this concept is realized as an extension of the CoAP protocol and Observe option. To illustrate this, we consider a simple smart lighting system that uses a light switch (the sensor) as a sensor, triggering a light bulb (the actuator) whenever pressed. This association may be made or modified at any time without the need for complete reconfiguration. We will use RESTful web services for the application and, represent the sensor resource by /gpio/btn and the actuator by /lt/on following the IPSO naming convention [17].

**Figure 4:**
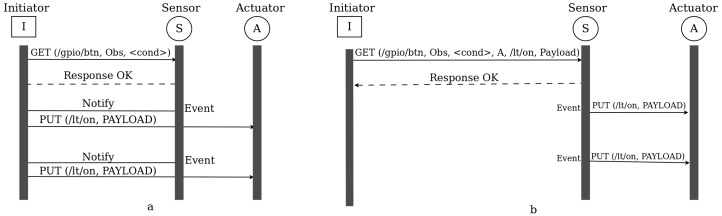
Sensor-Actuator Interaction. (**a**) Indirect; (**b**) Direct Binding.

In traditional intermediary-based solutions, all activities are coordinated by an intermediary device. As shown in Figure 4a, the communication begins by establishing an observation relationship between the intermediary, labelled Initiator, and the sensor. After that, whenever there is an event that results in a state change of the button resource (i.e., whenever there is a button press), the sensor sends a notification to the intermediary. Upon receiving the notification, the intermediary triggers the actuator. This can be even applied to dimmers where the light intensity of the bulb is related to the value sent from the switch as payload during notification. As can be seen in the figure, the intermediary must be always available for the system to work.

On the other hand, using the flexible direct binding approach that we propose, the initiator is only required to establish the relationship between the sensor and the actuator. After successful establishment of the binding relationship, the initiator is no longer required to take part in any of the subsequent communication between the sensor and the actuator (Figure 4b). This means, the initiator could be any IP capable device, such as a smart phone, that will just establish a relationship and then leaves and re-enters the network anytime. This relationship exists as long as the sensor and actuator are functional or until we exclusively change the binding.

The existing CoAP protocol does not support establishing binding relationship between two devices through a third device. When a device sends an observe request to another device, the relationship that will be established is between the sender and the receiver. A mechanism that allows the receiver to differentiate between a binding request and a traditional observation request needs to be introduced for our solution to work. In addition to this, some details of the actuator have to be communicated along with the binding request. In line with this, we modified the CoAP protocol by introducing four additional options, namely BIND_URI_HOST, BIND_URI_PORT, BIND_URI_PATH and BIND_PAYLOAD. BIND_URI_HOST and BIND_URI_PORT (optional in case default port is used) are defined to indicate the IP address and port number of the device that needs to be notified when events arrive. BIND_URI_PATH is the resource representation on the device through which it is triggered. When the request is stored on the sensor, the presence of these options is used to differentiate between a binding relationship and a traditional observation relationship. The optional BIND_PAYLOAD option may be used in a request if we wish a specific value to be sent during notification. In its absence, the new resource representation will be sent to the actuator. The method used for the notification will be PUT.

**Figure 5:**
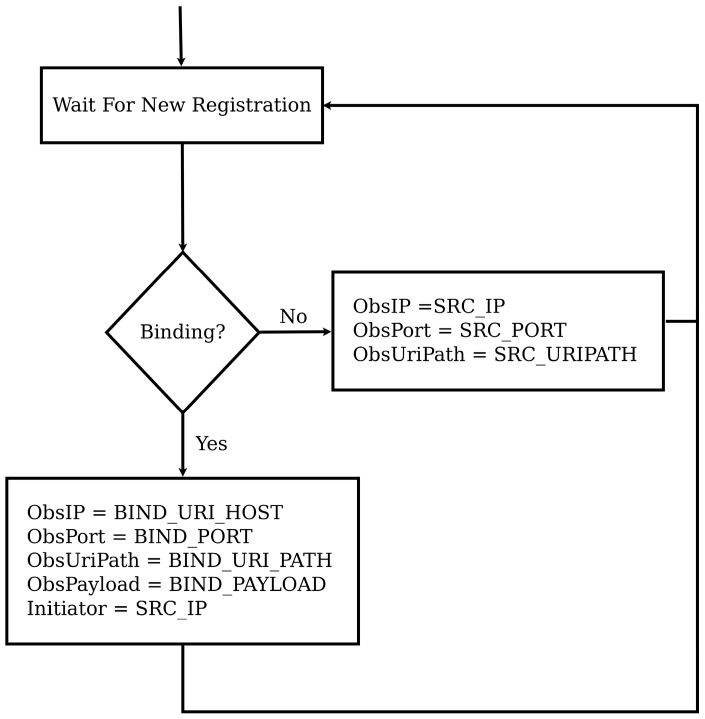
Flow Chart Showing Binding Relationship Establishment.

**Figure 6:**
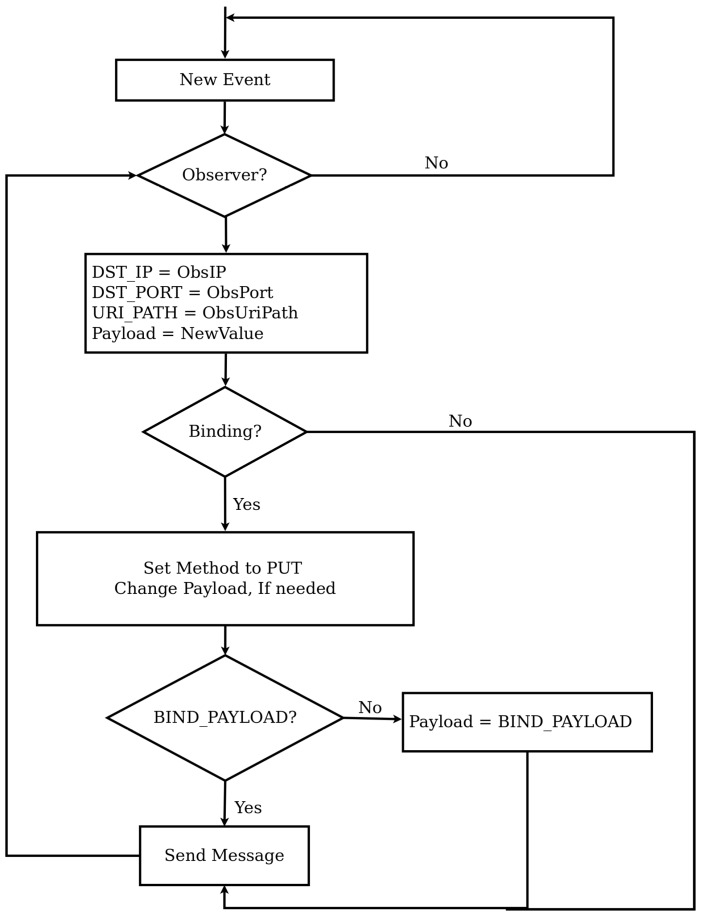
Flow Chart Showing Notification of Events.

Figure 5 shows the flow chart that describes how a binding relationship is established while Figure 6 shows the notification process. As shown in Figure 5, when a new request from the initiator that contains the observe option arrives at the sensor, it checks for the presence of one of the newly defined options to identify whether the request is a binding request or not. If so, the information contained in the newly defined options will be used as details to define the observer. If not, the source IP address, source port number and source URI_PATH will be taken as IP address, port number and URI path of the observer. For binding requests, we may optionally store the address of the initiator too. Once this is done, the initiator will not be involved in any event notification communications.

Referring to Figure 6, event notification of both binding and observation relationship is conducted in a similar way. The notification process starts when the state of an observed resource changes, which causes a packet to be sent to all observers. The notification process is almost the same for both binding and observation relationships except two steps. The first difference is the method. In case of a normal observation, a response packet is created while in case of a binding a PUT message is prepared. The payload to be used for binding relationships can be modified by the BIND_PAYLOAD option, which is the second difference. Apart from these two differences the notification process is the same.

Management of the binding relationships can be facilitated by introducing binding directories. Binding directories are similar to resource directories but list existing binding relationships. Through the binding directory, we may find out which binding relationships exist and perform reconfiguration by sending other binding requests, if necessary.

## 5. RESTlets

More and more IoT applications are moving into the Cloud. Many others reside at the gateway running proprietary protocols. Both options require movement of all data generated by sensors to the edge of the LLN or to the Cloud. As discussed in the previous section, this approach puts heavy burden on the border devices and some of the data that traverses the LLN might not be useful for the IoT applications. Performing some (pre) processing activities inside the constrained network can help to reduce the number of packets transmitted to the cloud or the edge.

**Figure 7:**
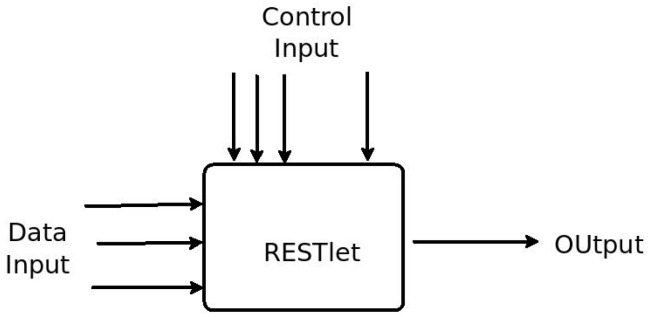
RESTlet Block Diagram.

In this section we will introduce a novel solution that breaks down IoT applications into smaller and manageable units in order to simplify IoT application development and resolve the problems mentioned above. The solution is based on what we call RESTlets. RESTlets can be defined as IoT application building blocks that use RESTful web services to process data inside the constrained network. RESTlets have one or more data and control inputs, processing logic and outputs (Figure 7). Internal wiring connects the data inputs, control inputs and the processing logic to generate new outputs. In the context of sensor/actuator interactions the data inputs could be sensor readings or outputs of other RESTlets. The processing that will be done inside the RESTlet may vary depending on the requirement of the application. The processing could be as complex as sending an SMS or as simple as a logical AND. If the processing results in new data, the output could be used to trigger an actuator or sent to another RESTlet as an input. The control inputs are configuration parameters that can be used to control how the RESTlet operates and how or what outputs must be produced. For example, the control parameters may define the threshold value for generating new outputs, or even the computation interval. By modifying the control parameters during runtime, we can control how a specific RESTlet behaves. After a RESTlet is defined on a specific device, it can be instantiated multiple times.

Implementation of RESTlets using RESTful web services can be achieved by representing each RESTlet instance as a resource and each component (data input, control input and output) as sub-resources. Instantiation of RESTlets is achieved by sending POST requests to the device that is selected to host the RESTlet. Since multiple RESTlets can be defined on a single device, the POST request must contain the name of the RESTlet along with the number of data inputs, control inputs and outputs. Whenever a POST request is received, the RESTlet resource and its sub-resources will be created dynamically and will be referenced using hierarchical naming convention as:

***/r/<RESTletNo>/<in|out|con>/<SubResourceNumber>***

For instance, /r/0/in/0 refers to the first data input of the first RESTlet while /r/1/out/0 and /r/0/con/1 respectively refer to the first output of the second RESTlet and the second control input of the first RESTlet. Please note that RESTlets are numbered as per the sequence of the POST request. This means the first RESTlet is the one created by the first POST request and so on. Alternatively, we can name them by passing the RESTlet name with the request for creation.

**Figure 8:**
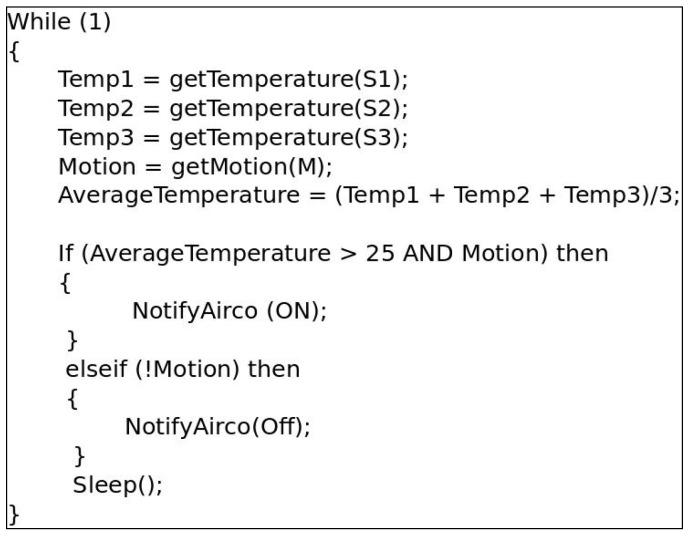
Sample Code Executed on Non-constrained Devices.

The combination of the RESTlet concept and the flexible direct binding concept can be used to build simple web-based IoT applications. To show how this works, we consider a simple smart home application that turns on or off the air conditioner based on the average temperature in an occupied room. The temperature values are obtained from three temperature sensors and occupancy of the room can be identified by a motion sensor. In traditional Web based IoT applications, the data from the sensors is sent to the LLN gateway to be processed there and the result will be sent back into the LLN for the actuator (in this case attached to the air conditioner) to act upon it. The data transmission from the sensors takes place in various ways. One possible way is by periodically polling for new values (shown in Figure 8). This would result in a lot of unnecessary data transfers in case the frequency at which temperature values change is much lower than the frequency of the polling interval. Another option would be to establish an observation relationship between the gateway and each sensor so that only new changes are communicated to the gateway. In both cases, data from all sensors is transferred to the gateway and triggers for the actuator, if any, have to be sent back into the LLN.

**Figure 9:**
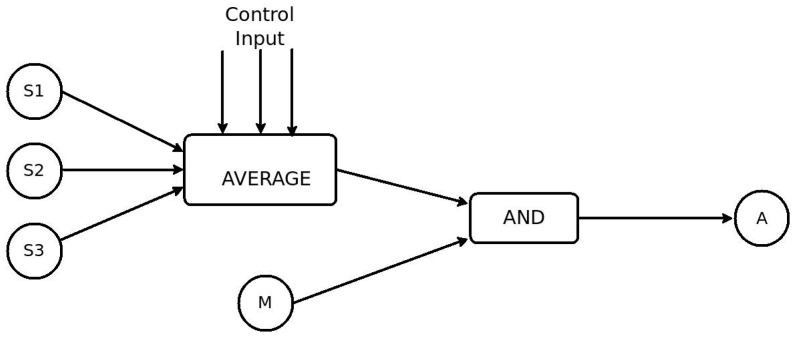
RESTlet block diagram for the smart home scenario.

**Figure 10:**
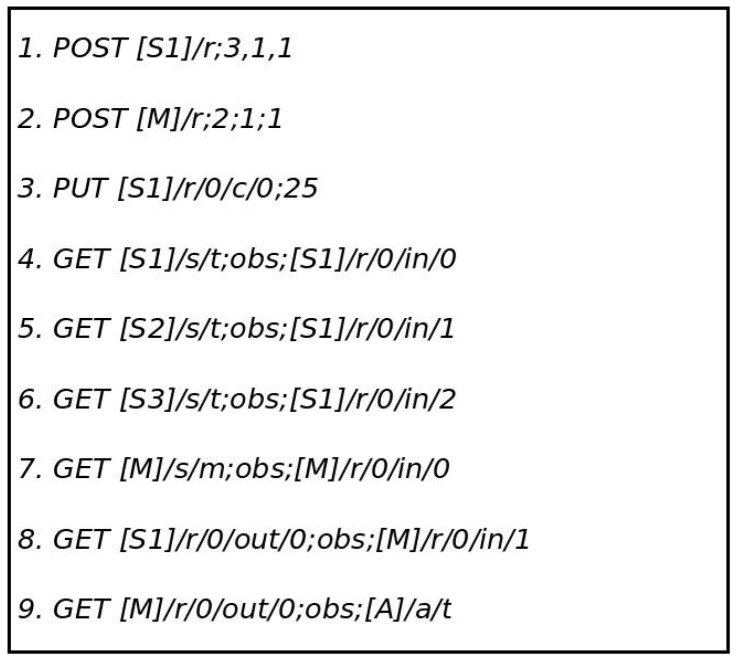
CoAP Messages used to create the required Binding Relationship.

Using RESTlets we can move some or all of the processing logic inside the LLN to reduce the number of packets transmitted. A block diagram showing the breakdown of the application into RESTlets is given in the diagram (Figure 9). The three temperature sensors send their data to the RESTlet which implements the AVERAGE function. The output of this RESTlet is sent to another RESTlet which implements the logical AND operator that combines this with the readings of the motion sensor to finally send the trigger to the actuator. One of the benefits of the RESTlet approach is that, any of the existing sensor or actuator nodes can be used to host the RESTlets. Alternatively, we can distribute the RESTlets among different nodes. Yet another alternative may be placing a more capable node inside the LLN that does all the processing. For simplicity, lets select node S1 to host the AVERAGE RESTlet and the motion sensor, M, to host the AND RESTlet. The figure below (Figure 10) shows the nine steps that can be used to program this application.

As shown in the above listing, the first two messages create the AVERAGE and the AND RESTlets on nodes S1 and M, respectively. The numbers indicate the number of data inputs, control inputs and output in that order. In this case, the AVERAGE RESTlet contains 3 data inputs while the AND RESTlet only has two. Both RESTlets have one control and one output each. The third statement sets the control parameter of the AVERAGE RESTlet to be 25 so that only average values greater than 25 °C are sent as output. Statements 4 through 6 establish binding relationships between each temperature sensor and the three data inputs of the AVERAGE RESTlet. After receiving these messages, the sensors send all temperature changes to the RESTlets data inputs. The seventh statement associates the motion sensor to the first input of the AND RESTlet by establishing a binding relationship between them. The output of the AVERAGE RESTlet and the second data input of the AND RESTlet are conveniently associated through observation relationship by the eighth statement. The last statement finally associates the output of the AND RESTlet to the actuator so that changes at the output will trigger the actuator. For the binding process to work properly, all outputs of RESTlets are made observable.

Interestingly, this simple concept reduces the whole IoT application development to a series of CoAP message transmissions that may be sent from anywhere in the network or over the Internet. Management of the IoT applications is also made easy. Sending simple GET messages to the RESTlet nodes and using binding directories to list out all available bindings gives us enough information to inspect and, if needed, reprogram the entire application or to modify some aspect of it.

## 6. Implementation and Evaluation

### 6.1. Implementation

The selection of a good implementation platform is crucial to demonstrate the feasibility of new concepts and to show performance gains obtained through the proposed solutions. We used Contiki 2.7 [18] as a base system for all experiments, which was the latest stable version available at the time of starting our experiments. Contiki is an open-source embedded operating system suitable for constrained systems. Its innovative IP implementation, uIP, makes it a good solution for experiments that involve IP-based communications in the constrained world. In addition to this, all required features for our tests such as 6LoWPAN, RPL, and CoAP have all been implemented. The CoAP implementation of Contiki is known as Erbium [19].

Next to this, we also need to run some tests on non-constrained devices for comparison purposes. This requires a CoAP implementation that runs on non-constrained devices (e.g., gateways, Cloud servers, etc.). For this, we have used our own C++ based implementation of CoAP and its extensions, named CoAP++. Since both Erbium and CoAP++ do not support the proposed binding and RESTlet concepts, some modifications have been made, including the addition of new CoAP options and the introduction of a mechanism to define and instantiate RESTlets.

#### 6.1.1. Flexible Direct Binding

As explained earlier, to support the binding concept, four new options were introduced. These new options have been added to Erbium and CoAP++ with the following option numbers 42, 46, 50, and 54 for BIND_URI_HOST, BIND_URI_PORT, BIND_URI_PATH and BIND_PAYLOAD respectively. In addition to this, the required functionality for serializing and parsing those options has been added. Apart from this, two other modifications have been made in order for the binding solution to work properly. The first major modification included an extension of the registration mechanism in order to differentiate between normal observers and binding observers as shown Figure 5. The second major change was the way notifications were sent to observers, as for binding relationships, the PUT method is being used, optionally in combination with a payload as indicated by the BIND_PAYLOAD option.

#### 6.1.2. RESTlets

The RESTlet concept, as discussed above, makes use of bindings to build (parts of) IoT applications by performing processing tasks and exchanging raw values and (semi) processed data between devices. In order to enable nodes to support RESTlets, some modifications have been made to both Erbium and CoAP++. The main modifications are discussed below.

**Figure 11:**
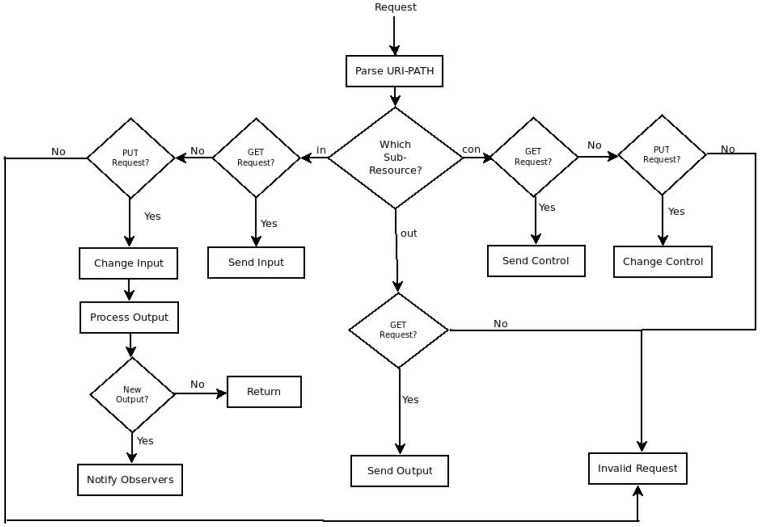
Flowchart Showing Interaction with RESTlet Instances using CoAP Messages.

The application logic of RESTlet, which acts on the data inputs (and the control inputs) to produce outputs, has been implemented for every node that potentially hosts the RESTlet. Every RESTlet defines its own processing function and hence one processing function per RESTlet is defined. Generic functions that manipulate the data and control inputs have also been defined. A POST request to a specific resource initiates the instantiation of the RESTlet instances and the dynamic creation of associated resources. The /r resource is defined for this purpose. Moreover, since Erbium does not support the dynamic creation of resources, this functionality has been added. In order to differentiate one RESTlet instance from the other, detailed information about every instantiated RESTlet needs to be stored. Therefore, a data structure for storing the RESTlet name, the number of data inputs, the number of control inputs, the number of outputs, and the memory address of the processing function has been defined. This data structure may also store the latest values of the data inputs, control inputs and outputs if required. Finally, a callback function that is called for further manipulation of the resources and sub-resources has been defined. The flow chart of this callback function is shown in Figure 11.

Each time a request for the */r* resource arrives, it is forwarded to the callback function in order to see which sub-resource is referenced and an appropriate action is taken. Based on the URI-PATH of the request, the function will be able to identify for which sub-resource the request has been sent. A PUT request for the data input (in) initiates execution of the processing logic, which, in turn, notifies observers in case the output changes. Sending a PUT request to the control input (con) results in changing the identified control input. GET requests to any of the sub-resources may be used to retrieve the current value. All outputs have been made observable in order to allow binding relationship between the RESTlet and devices (or other RESTlets).

Putting it all together, once all logic for a particular RESTlet has been defined and implemented, applications may instantiate RESTlets on a specific node by sending a POST request to the */r* resource and specifying the name of the RESTlet, the number of data inputs, control inputs and outputs in the payload as follows:

***RN* = <*RESTlet Name*>; *IN* = <*# Data inp*>; *CON* = <*# Control inp.*>; *OUT* = <*# Output*>**

For example, a POST request sent to a node with RN = AND; IN = 2; CON = 2; OUT = 1; as payload, creates the AND RESTlet with two data input, two control input and out output resources. The five resources will be referenced as /r/0/in/0, /r/0/in/1, /r/0/con/0, /r/0/con/1, and /r/0/out/0 in all further communications. Subsequent POST requests create RESTlet resources that are identified by changing the number next to the */r*.

### 6.2. Experiment Setup

For all experiments involving constrained nodes, we simulated Zolertia (Z1) nodes in Cooja. The basic scenario we tried to simulate is the interaction between a temperature sensor (as sensor), identified by the /s/temp resource, and a thermostat (as actuator), identified by /a/t. The temperature values are periodically read from a random sequence of 100 values stored in an array. If two consecutive readings result in different values, a notification to the observers will be sent. Whenever a non-constrained node is involved, we use the CoAP++ code running on the laptop.

### 6.3. Functional Evaluation

In this subsection the details of the proposed solutions and their implementation are discussed.

#### 6.3.1. Bindings

**Figure 12:**
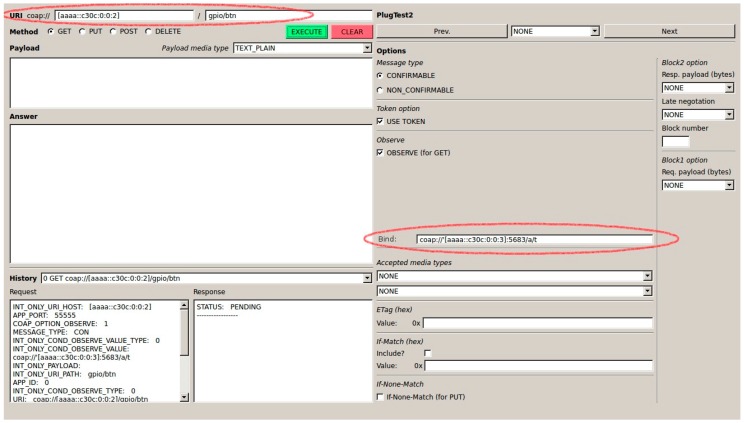
Creation of Binding Using CoAP++ GUI from Non-constrained Device.

As explained earlier, binding a sensor and an actuator can be done by any device from anywhere in the Internet. Figure 12 shows the CoAP++ GUI screenshot when a GET request is used to establish a binding between the /gpio/btn resource on a sensor with IP address [aaaa::c30c:0:0:2] and an actuator with address [aaaa::c30c:0:0:3]. The specific resource of interest on the actuator is /a/t.

**Figure 13:**
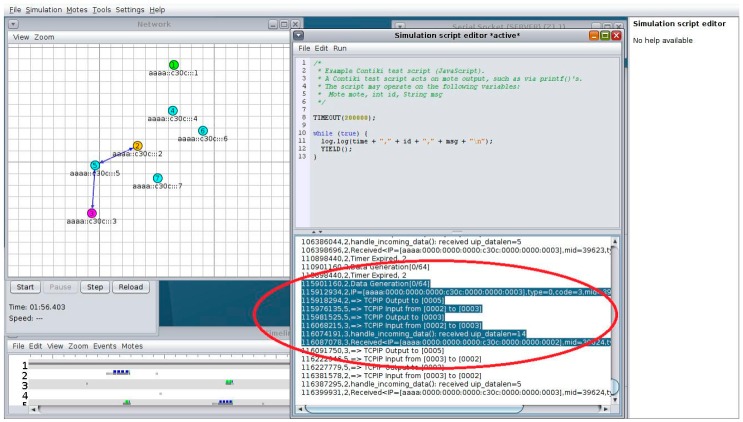
Direct Interaction of Sensor and Actuator Nodes in Cooja.

Once the binding relationship has been established, all further interactions take place directly between the sensor and the actuator. One such an interaction can be seen in Figure 13, which is Screenshot of the Simulation in Cooja. The Cooja Visualizer at the left of the picture shows the direct communication (the notification and the ACK) in blue arrows. The shaded part of the simulation script editor window confirms the route the packets followed after data is generated at the sensor (Node 2) until received by the actuator.

#### 6.3.2. RESTlets

**Figure 14:**
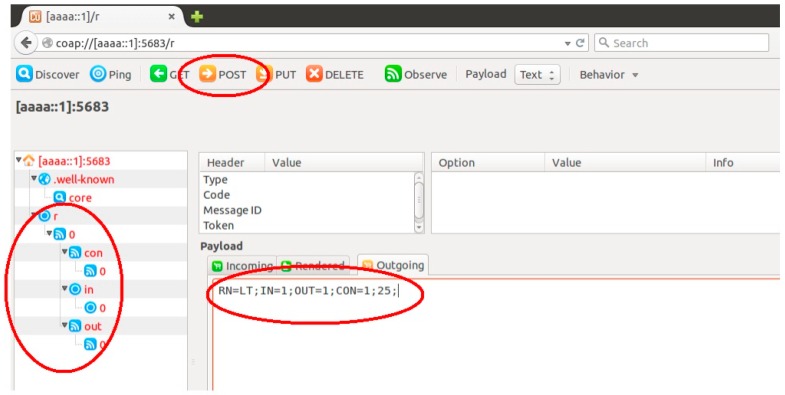
Creation of RESTlet Instances in Copper.

RESTlets are application building blocks that may be defined once on devices and that can be instantiated a number of times to build (part of) IoT applications by interconnecting them with devices and each other using flexible bindings. This process can also be accomplished using any device connected to the Internet. To illustrate this, we consider the example of a simple LESS-THAN RESTlet in order to send notifications to the actuator shown above in case the temperature drops below 25 degrees. Figure 14 shows the Copper screenshot of this operation. Sending a POST request to the */r* resource of the node selected to host the RESTlet, in this case [aaaa::1], creates the resource and its sub-resources. The payload shows the name of the RESTlet, LT indicating less than, which has one data input and one control input. The control input is initialized to 25. The default number of outputs is 1. Upon reception of the POST request the RESTlet is being instantiated and further referenced as /r/0. In addition, 3 sub-resources identified as /r/0/con/0, /r/0/in/0 and /r/0/out/0 are created representing the control input, data input and output, respectively.

To achieve the desired result, the input /r/0/in/0 is bound to the sensor node and the output /r/0/out/0 is bound to the actuator. This way, sensor readings are being transmitted to the RESTlet and the output of the RESTlet triggers the actuator. For this, the binding functionality shown in the previous sub-section is applied twice. Subsequent updates of the control parameter can be easily performed by sending a PUT request to the /r/0/con/0 resource.

This solution can be used to build complex IoT applications by distributing the RESTlets at different devices inside the LLN, at the LLN Gateway or even in the cloud. Irrespective of the complexity of the application or the location of the RESTlet nodes, we send a series of CoAP requests to the devices to program the application. To simplify the development even further, we can employ visual programming tools to simply drag-and-drop components to instantiate RESTlets and perform the binding.

### 6.4. Performance Evaluation

The proposed modifications may affect some aspects of the network or the device itself. Memory footprint, number and size of packets transmitted, and communication delay are some of the parameters that might be affected positively or negatively. The outcomes of several tests showing these impacts are discussed below.

#### 6.4.1. Performance Evaluation of Bindings

##### A. Memory Footprint

As described above the original Erbium code has been modified in order to support the binding concept. This modification induces a slight increase in memory space, mainly in the code (text) segment. Table 1 shows the increased memory footprint of the binding solution compared to gateway or cloud-based solutions. As every observer’s information needs to be stored in memory, the memory required in the BSS section increases proportionally to the number of observers. However, the difference between the two approaches is only 38 bytes per observer for the two cases.

Despite the slight increase in memory footprint, the code can still fit in constrained devices. Given the advantage of the binding solution, the increase in memory footprint is acceptable and the binding solution is viable to be applied in constrained devices. However, this does not come without a limitation. An increased number of bindings leads to an increase in memory space requirement. The BSS section of both solutions in the table shows that when the number of observers increases, the size of the BSS increases as well because at boot time the program always reserves the maximum amount of memory needed to store all potential observers. As memory is a very scarce resource of constrained devices, this will limit the number of observers allowed to register simultaneously and thus the number of bindings that can be supported. Here the gateway/cloud solution has an advantage since it may achieve scalability by aggregating multiple observe requests at the gateway avoiding one to one relationships between multiple actuators and a sensor.

##### B. Packet Size

LLNs have a low Maximum Transmission Unit (MTU). Large packets whose size exceeds the MTU go through a fragmentation/defragmentation cycle from the source all the way to their final destination. This behaviour negatively affects the performance of the network. Therefore, the resulting packet size is a very important parameter when discussing the performance of new solutions. Moreover, fragmentation also comes at the expense of an increased delay. The packet size at the application layer for CoAP based communication can be calculated as:

Packet Size = Size of (CoAP-Header) + Size of (Token) + Size of (options) + Size of (payload)

where:

Size of (CoAP-Header) = 4 bytes, Size of (Token) = 0 to 8 bytes

Size of (Options) differs from packet to packet depending on the number and type of CoAP options being included in the packet. For example, Observation requests include the Observe Option, which has a maximum length of four bytes. The Uri-Path option and payload greatly vary depending on the resource identifier and the data to be communicated. For the URI path, we assume the simplified IPSO Application Framework [17] resource names. For instance, for a button associated with a light switch (sensor)the URI path becomes /gpio/btn, which will be transmitted as two Uri-Path options with a total length of nine bytes (one byte for every option plus the length of both segments “gpio” and “btn” in the URI).

Most of these values are common for all types of communication so they do not impact the comparison between the two methods. The real difference between the two solutions can be seen at the relationship initiation packet. In case of the non-binding solution the options that are minimally needed are Observe (one byte) and Uri-Path (nine bytes for /gpio/btn). Including the CoAP header (four bytes) and the token (one byte in this example), the total packet size will be 15 bytes. However, for direct bindings, the initial packet includes four additional binding options containing the information on how to trigger the actuator. Therefore, the number of additional bytes required, B_Byte_ is given by:

B_Byte_ = Size of (BIND_URI_HOST) + Size of (BIND_URI_PORT) + Size of (BIND_URI_PATH) + Size of (BIND_PAYLOAD)

where:

Size of (BIND_URI_HOST) = O + 16 /*IPv6 address*/

Size of (BIND_URI_PORT) = O + 2 /*Optional. Default CoAP Server port is used*/

Size of (BIND_URI_PATH) = Sum of (O + size of (path_segment i))

with i going from 1 to # of path segments:

Size of (BIND_PAYLOAD) = O + X

In the above formula, O is the number of bytes needed for encoding the option delta and option length (between one and five bytes, but one in most cases). The value X depends on what we want to transmit in the payload. In our example, we send a single byte information and hence X is equal to 1. Further, we assume the actuator uses the default CoAP server port. Using this formula, the additional number of bytes required for our example is given by B_Byte_ = 19 + 6 + 2 = 27 Bytes. Considering the 15 common bytes, the total packet size for the binding solution will be 42 bytes. Even if the packet size of the binding solution is bigger than the one of the gateway-based solution, it does not affect the network performance at all. First, this request is sent only once in order to establish the relationship. Once the binding has been established, there is no further communication of this size. Had it been the packet size of the notification, it would, indeed, impact the network negatively. In addition, the packet size is yet in the limit of the LLNs MTU, being 127 bytes at the MAC layer. Hence, no fragmentation will be applied that negatively affects the network performance.

##### C. Communication Delay (Latency)

**Figure 15:**
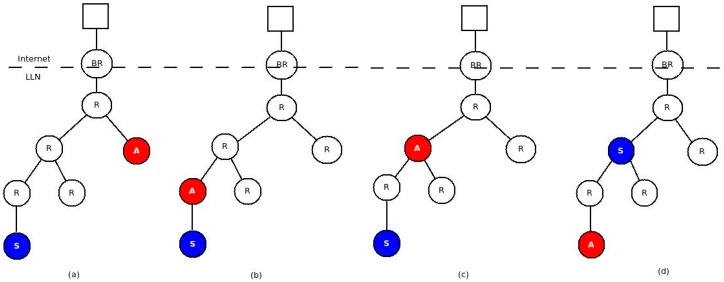
Topologies: (**a**) Sensor and actuator in different branch of the tree; (**b**) Actuator between Sensor and Gateway—directly connected; (**c**) Actuator between Sensor and Gateway after 1 hop; (**d**) Sensor between Actuator and Gateway after 1 hop.

Delay is an important parameter to compare the performance of different solutions. The route packets take to reach destination plays an important role in determining the communication delay. The route, in turn, depends on the network topology. Therefore, we need to consider different topologies to compare latencies between the two approaches. In this experiment, we considered four topologies as shown in Figure 15.

**Figure 16:**
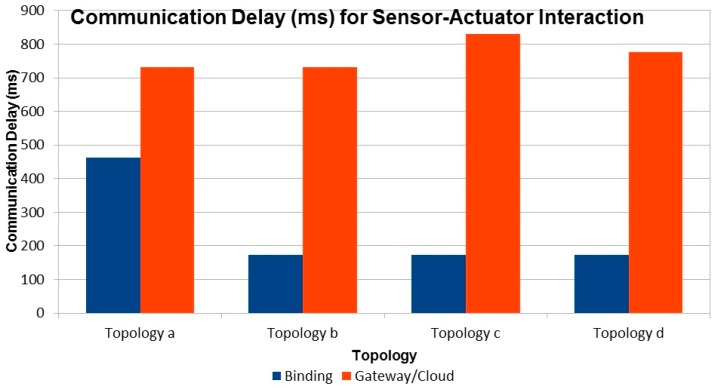
Communication Delay (ms) vs. Topology.

For all topologies the latency is computed as the time difference between the occurrence of the event at the sensor and the reception of the PUT packet by the actuator. From the results depicted in Figure 16, we can see that in all cases the gateway/cloud based solution has a significantly higher latency compared to the binding solution. This is expected as all sensor events are sent all the way to the gateway and triggers come down to the actuator in the non-binding solution. This increased number of hops introduces significant delay in the overall notification/trigger cycle. The delay will be even more pronounced for larger networks.

**Figure 17:**
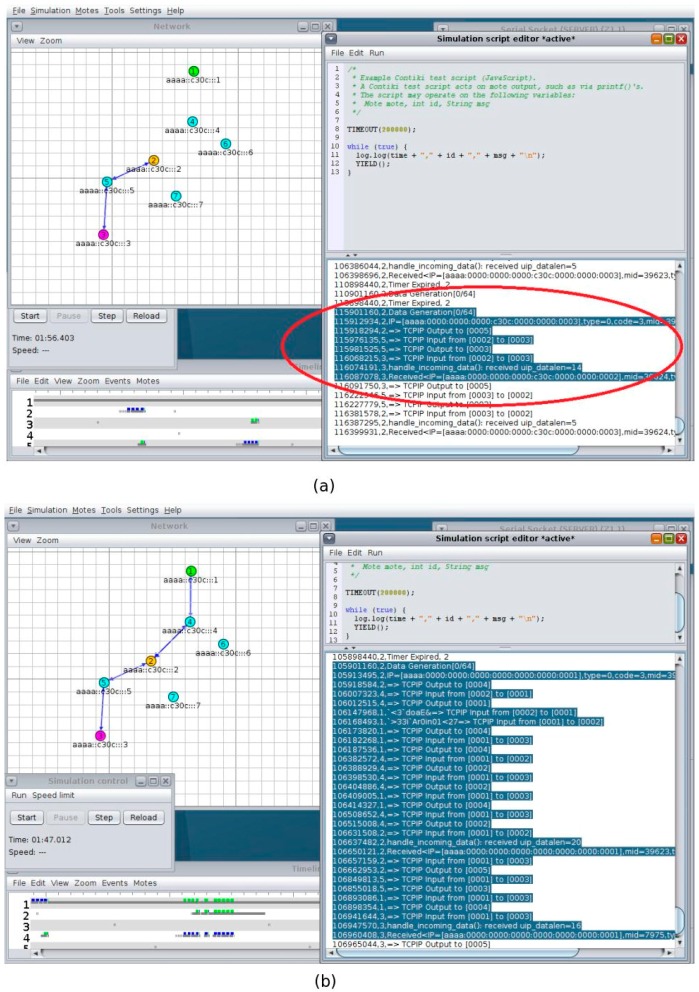
Sensor-Actuator Interactions. (**a**) Binding (**b**) Gateway/Cloud-Based Solution.

For our solution, the number of hops, and hence the delay, depends on the routing protocol. As we mentioned earlier, we used RPL as routing protocol. In RPL, the furthest the packets travel is until the common parent of the sensor and actuator. The closer the sensor and actuator, the less delay is introduced. The Cooja screenshots (Figure 17a,b) confirms this statement. The blue arrows in the left of Figure 17a shows that the interaction is direct between node 2 and 3 while that of Figure 17b shows that the interaction goes through the border router. The shaded part in the right shows the route the packets take from the sensor to its ultimate destination (the actuator).

##### D. Number of Packets 

An increased number of packets in constrained networks lead to an increased power consumption at each router node and more delay. Therefore, looking at the number of packets generated by the two solutions that strive to achieve the same goal is a good performance measure to compare both solutions. As every notification goes through the gateway, the gateway-based solution creates one additional packet for every notification. If the packets are sent as confirmable requests, this number will be doubled. As the number of sensors and actuators increases, the number of packets generated will also increase significantly. In dynamic systems where notifications are generated frequently, the number of packets being generated gets higher and higher. 

#### 6.4.2. Performance Evaluation of RESTlet

Several tests were conducted to evaluate the performance of RESTlets. In all tests, we considered latency to be the most important performance factor that needs to be compared. We used different topologies, number of nodes, and data processing entities.

##### A. Impact of RESTlets

**Figure 18:**
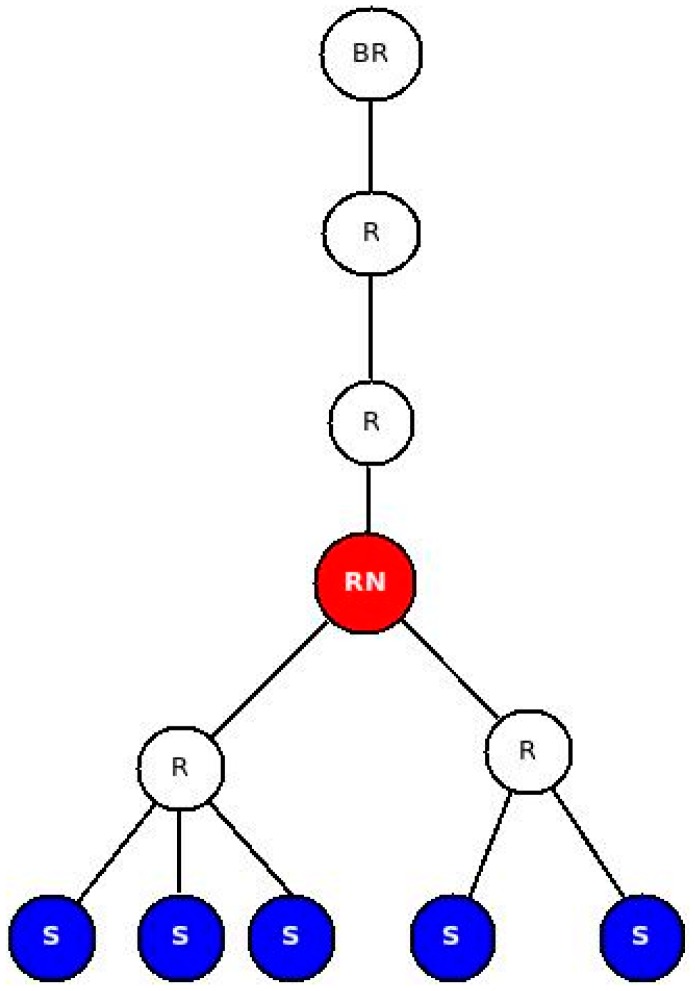
Network Topology.

Data processing performed in the LLN by RESTlets introduces delay but reduces the number of packets in the network. We used a fixed topology (Figure 18) to mathematically evaluate the impact of RESTlets at the RESTlet node (labeled RN in the figure). In this scenario, data may be sent from the sensor nodes, labeled S, and pass through the RESTlet node before going out to the LLN gateway or the cloud. We compared the RESTlet case, where processing is done by the RESTlet node and No-RESTlet case, where the processing is done elsewhere (at the gateway or in the cloud). In the No-RESTlet case, node RN is used as a router only. Whenever a packet arrives, it just processes it in order to determine the next hop address after which it is forwarded to the next hop.

Therefore, in the No-RESTlet case, the total packet processing and forwarding time at the node RN is given by:

*T_p_* = *T_x_* + *TF*_1_ + (*TD*_1_ + *T_x_* + *TF*_2_) + … + (*TD_n_* + *T_x_*)

where:

- *T_p_* is total packet processing and forwarding time.
- *T_x_* is packet processing time (from the experiment we found out that this value is 6 ms)
- *TD_i_* is time delta between the arrival of two consecutive data packets from two different senders (if only 1 data sender, this value is 0). This value is variable.
- *TF_i_* is Packet forwarding time (calculated as the arrival time of the packet at the next hop minus the time the packet was ready to be sent out). This value is also variable.

So for *n* data generating nodes;

$$T_{p}=\{\begin{array}{l} T_{x}+\;TF_{0},\;\;\;\;\;\;\;\;\;\;\;\;\;\;\;\;\;\;\;\;\;\;\;\;\;\;\;\;\;\;\;\;\;\;\;\;\;\;\;\;\;\;n=1\\ T_{x}\times n+\;\sum_{i=2}^{n}TD_{i}+\;\sum_{i=1}^{n}TF_{i},\;\;\;n>1 \end{array}$$

On the other hand, in the *RESTlet case*, the node is expected to do other processing too. First of all, it has to unpack the CoAP packet to get the data and store it provisionally. Secondly, it waits for subsequent packets if more than one data sender node exists. Thirdly, it has to perform processing and generate output. Finally, a new packet is generated and forwarded to the next hop. 

Therefore, the total packet processing and forwarding time at the RESTlet node, *T_p_*, is given by:

*T_p_* = *T_x_* + (*TD*_1_ + *T_x_*) + … + (*TD_n_* + *Tx*) + *T_NPG_* + *TF*

where:

- *T_p_* is total packet processing and forwarding time.
- *T_x_* is packet processing time. (From the experiment, we found out that this value is 22 ms and the RESTlet function we considered was AVERAGE. Other processing functions may yield different results).
- *TD_i_* is time delta between arrivals of two consecutive data packets from two different senders (if only 1 data sender, this value is 0). This value is variable.
- *TF* is packet forwarding time (calculated as the arrival time of the packet at the next hop minus the time the packet was ready to be sent out). This value is also variable.
- *T_NPG_* is time required to generate new packet (from the experiment we found out that this value is 14 ms).

So for n data generating nodes,

$$T_{p}=\{\begin{array}{l} T_{x}+\;T_{NPG}+TF,\;\;\;\;\;\;\;\;\;\;\;\;\;\;\;\;\;\;\;\;\;\;\;\;\;\;\;\;\;\;\;\;\;\;\;\;\;n=1\\ T_{x}\times n+\;\sum_{i=2}^{n}TD_{i}\;+T_{NPG}+TF,\;\;\;n>1 \end{array}$$

In order to compare both results, we can say that *TD_i_* is the same for both cases and can use an average constant number for simplicity. However, the value of *TF_i_* is different among different packet transmissions. From the experiments, we observed that the data forwarding interval ranges between 20 ms and 140 ms. Moreover, all experiments showed that the processing time at node RN, *T_x_*, is 6 ms and 22 ms for the No-RESTlet case and the RESTlet case, respectively. The new packet generation time, *T_NPG_*, for the RESTlet case was also found to be 14 ms. In order to see the difference in terms of processing time, we used the following simplified formula by using the aforementioned values as an average:

$$\text{For No-RESTlet case (for }n\text{ number of data generating nodes):}\;T_{p}=6ms\;\times \;n+TD+\left(TF_{i}\;\times n\right)$$

$$\text{For RESTlet case (for }n\text{ number of data generating nodes):}\;T_{p}=22ms\;\times \;n+TD+14ms+\;TF_{i}$$

**Figure 19:**
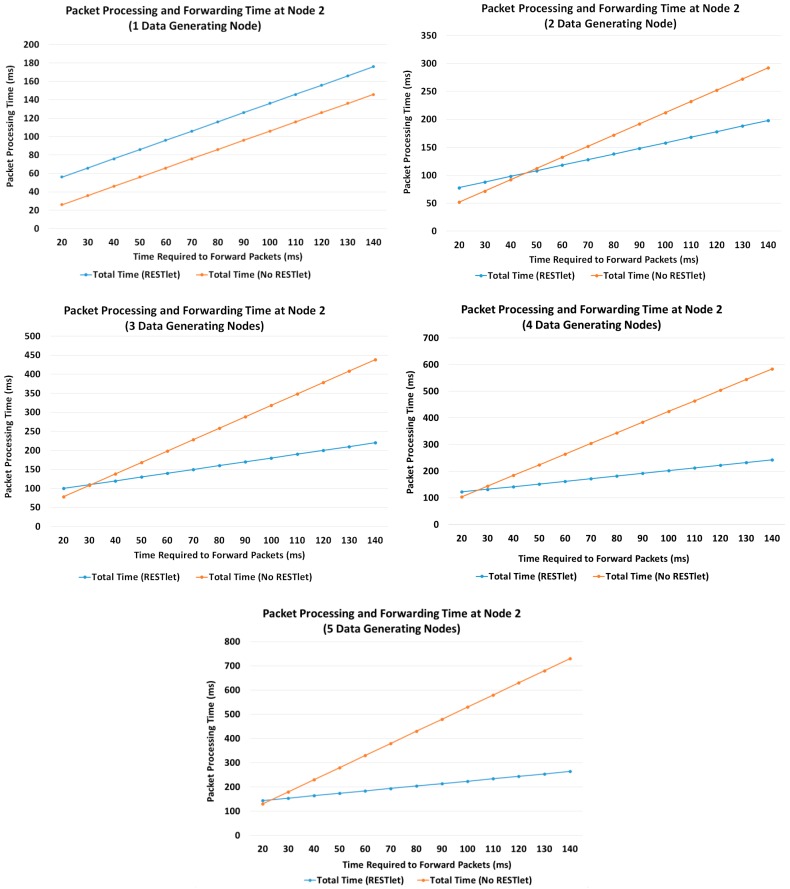
Packet Processing and Forwarding time at RESTlet Node for Various Number of Data Generating Nodes.

This enables us to calculate the resulted packet processing them for a varying number of data generating nodes (1, 2, 3, 4 and 5). Figure 19 depicts the result in graphs for both approaches using different *TF_i_* values.

Figure 19 shows that when the number of data generating nodes becomes more than one, the delay introduced by processing incoming packets by RESTlets becomes less important. For congested networks, which are characterized by larger *TF_i_* values, the advantage will become more pronounced. This is due to the fact that the RESTlets only generate a single packet after processing (or no packet at all depending on the type of processing) whereas the No-RESTlet case blindly forwards all the packets it receives which will be subject to large forwarding times.

##### B. End to End Latency with Multiple Data Nodes (Impact of Number of Nodes)

**Figure 20:**
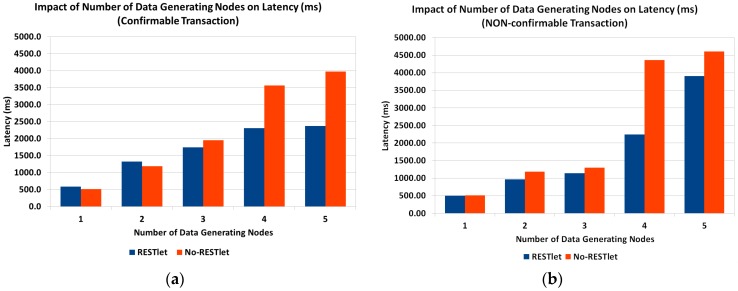
Impact of Number of Data Generating Nodes on End-to-End Latency. (**a**) Confirmable Communication; (**b**) NON-Confirmable Communication.

In the previous sub-section we mathematically showed that the reduced number of packets that results from the aggregation process by RESTlets compensates for the processing delay introduced by the RESTlets and result in better latency. To prove this concept, we measured the actual end-to-end delay from data generating nodes all the way to the border router. We used the topology shown in Figure 18 (above). The data nodes generate data every five seconds that will go to the border router. In the RESTlet case, all data is sent to the RESTlet node (RN) which processes the data and generates a new packet destined to the border router. The new packet is generated either upon arrival of data from all data nodes or within five seconds interval, depending on which condition is met first. The end-to-end latency is calculated as the difference between the data generation time of the first node and the arrival of the new packet at the border router. On the other hand, for the No-RESTlet case, all data is sent directly to the border router by traversing the RESTlet node as a router. In this case, the end-to-end latency is computed by taking the difference between the data generation time of the first data node and the arrival time of the last data packet at the border router. We run the tests by sending the packets as CONfirmable and NON-confirmable requests. Figure 20 shows the results.

For both CON and NON transactions, the RESTlet case results in a reduced latency compared to the No-RESTlet case. The difference is significantly higher for the confirmable case when the number of data nodes is higher. In the no-RESTlet case, all data packets are forwarded to the border router which is expected to produce ACKs for all. This results in an increased load on the border router and hence increased latency. Even if the processing is done by an external more powerful device such as the gateway, still all requests, acknowledgements and responses have to go through the border router and contribute to the increased overall latency of the NON-RESTlet case.

In the experiments we conducted, we made two interesting observations. When the transaction is CON, there were a number of duplicate packets and out of order arrivals especially when the data nodes are more than three. This is much more visible for the NO-RESTlet case where, out of 75 packets sent, there were 28 duplicate packets while for the RESTlet case there were only 13 duplicates when the number of data generating nodes is five. The other observation is the difference in packet loss between NON and CON transactions. As expected, the NON transactions suffer from packet loss in both RESTlet and NO-RESTlet cases with staggering 15% and 30% loss, respectively when the number of data generating nodes is four.

Finally, comparing the CONfirmable and NON-confirmable transmissions of packets, it is not surprising to see that the CONfirmable messages result in a higher latency as compared to NON-confirmable transactions. However, when there is large number of data generating nodes, the latency difference gets smaller for NON-confirmable transactions. The reason is the higher rate of packet loss forces the processing of packets to be made at the end of the five second interval.

##### C. Impact of Other Nodes

**Figure 21:**
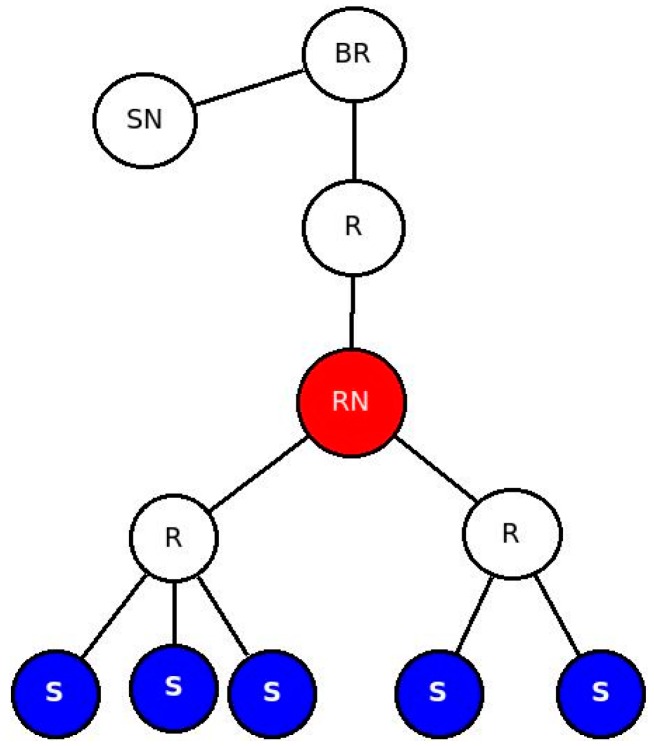
Network Topology including a Node Generating Side Traffic.

**Figure 22:**
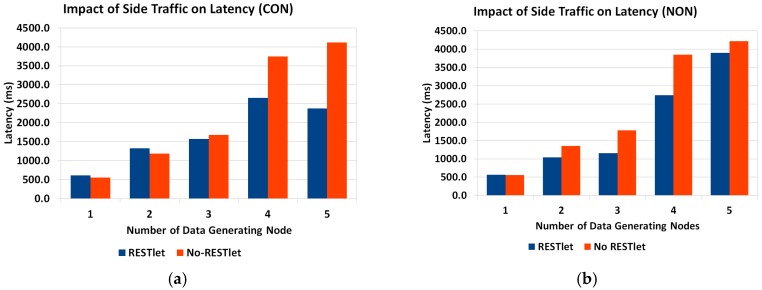
Impact of Side Traffic on Latency. (**a**) CONfirmable; (**b**) NON-Confirmable transaction.

Under normal working conditions, other communications may take place inside the LLN that may interfere with the interactions under consideration. To study the impact of such side traffic, we added another node that sends packets every 500 ms to the border router (Figure 21). The result is depicted in Figure 22. As expected, due to the additional packets at the border router, the latency has shown some increase.

##### D. Impact of Difference of Data Arrival Time

All the above tests showed that, the higher the number of packets that are being generated and transmitted inside the constrained network, the performance of both solutions, especially the NO-RESTlet solution, suffers. We run additional tests to observe the impact of the data arrival time difference on the latency by inserting an artificial gap in the data generation at the sensor nodes. All data generation nodes are made to generate data randomly between 0 ms and a maximum interval (this represent real world cases where multiple sensors observe the same physical phenomenon almost simultaneously). We used 500, 1000, 1500 and 2000 ms as maximum interval. The topology used is the same as the first test (Figure 18). We also run the experiment to observe the impact for number of data generation nodes.

**Figure 23:**
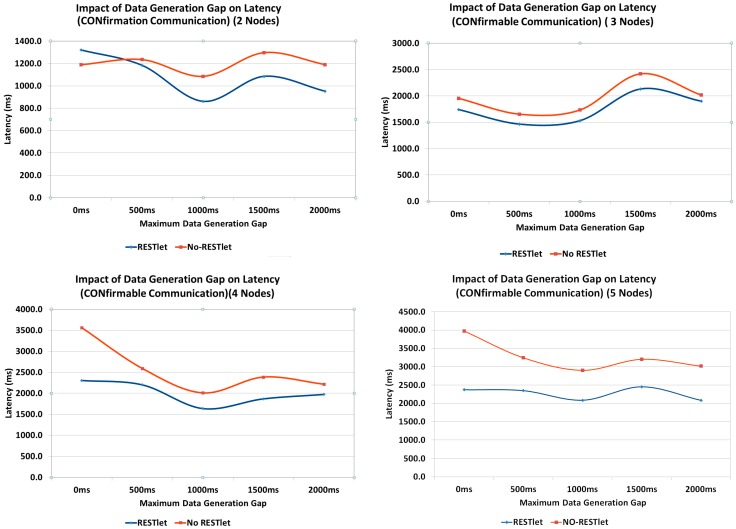
Impact of Packet Arrival Time Gap on Latency.

As can be seen in Figure 23, when there is no data generation gap (0 ms gap), the NO-RESTlet solution has much higher latency in most cases. The frequent arrival of packets at the border router creates congestion at the node. Due to the limited queue size of the constrained nodes, some packets will be dropped requiring retransmissions. This is the reason for the significantly high latency at 0 ms gap. When we look at the general trend in all graphs, the latency for both cases reduces until 1000 ms data generation gap and starts rising slightly after that. The reason is simple. The introduction of artificial delays at the sensor node results in an additional delay in the end-to-end transmission. This means, the performance gain obtained by separating the arrival times will be countered by the artificial delay and as a result the overall latency starts to rise.

##### E. Impact of Noise 

**Figure 24:**
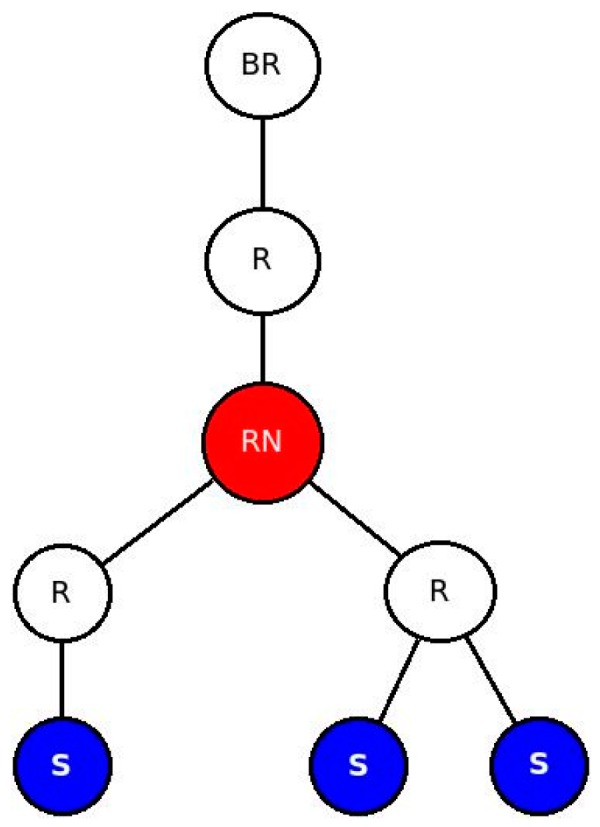
Network Topology for Noisy Networks.

Under normal working conditions, sensor and actuator nodes suffer from interference from other sources. This might create loss of packets requiring retransmissions in case of CONfirmable transmissions which, in turn, leads to increased latency. NON-confirmable transactions also suffer from increased latency since every lost packet leads to processing to be delayed until the 5 s interval is reached. To study the impact of lossy networks on latency, we run tests by setting the Transmission/Reception (TX/RX) loss from 0% (no loss), 5% and 10% losses. The topology we used is given in Figure 24. As the figure shows, there are three data generating nodes that send packets every five seconds without any time gap between the data generations. We selected three nodes to avoid the impact of having too many or too few data nodes which might skew the result to either side. Too little data generating nodes may influence the result in favor of NO-RESTlet case while too many nodes favor the other. The test is done both for CONfirmable and NON-Confirmable transactions.

**Figure 25:**
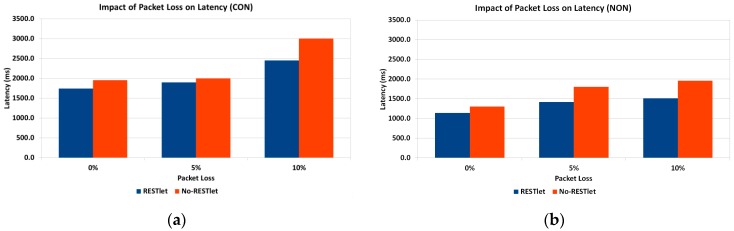
Impact of TX/RX Reception Ratio on Latency. (**a**) CONfirmable Communication; (**b**) NON-Confirmable Communication.

It is not a surprise that the results of the experiments in Figure 25 show higher overall latency for the NO-RESTlet case in both CON and NON communications. However, it is quite interesting to see that the difference between the NO-RESTlet and the RESTlet cases gets higher at higher TX/RX loss ratios. This indicates that, our solution is relatively more robust under lossy conditions for both CON and NON transactions.

## 7. Conclusions and the Way Forward

In this paper we presented two novel concepts that simplify sensor and actuator interactions and IoT application development by leveraging on CoAP as a protocol in combination with the Resource Observation extension. The binding concept effectively enables flexible direct interactions between sensors and actuators making gateway/cloud based solutions where intermediary devices accept input from sensors in order to trigger actuators redundant. The proposed solution, reduces the packet flow to the gateway and hence reduces latency and number of packets in the LLN compared to gateway or cloud based solutions. Through experiments we showed that the overhead (e.g., memory footprint) introduced by the binding solution is not significant compared to the gateway/cloud based solutions. In fact, regarding many aspects such as communication delay and number of packets, the binding solution outperforms traditional solutions. We also showed that this flexibility can be achieved by only making minor changes to the CoAP protocol and the observe extension. 

The other novel concept, RESTlets, builds upon this binding concept. RESTlets are IoT application building blocks with data and control inputs, processing logic and data output. We showed that by using RESTlets as IoT application building blocks, we can do in-network processing and aggregation in order to reduce the number of packets that traverse the whole LLN to the edge of the network and/or to the cloud which otherwise would lead to higher latency. We also showed that by interconnecting the data inputs and outputs of RESTlets to sensor outputs, actuator inputs or other RESTlets, we can build a complete IoT application within the LLN. Since the RESTlet approach allows distributed deployment of the processing logic at different nodes, there will not be too many resource hungry processes on one single node. It also gives greater flexibility in developing IoT applications by placing simple processing functionality inside the LLN and more complex one at the gateway or in the cloud. We ran several experiments in order to evaluate the performance of our solution by comparing it to traditional gateway-based or cloud solutions by using a different number of data generating nodes, data generating gap and TX/RX ratio. In all cases, our solution is capable of outperforming traditional solutions in terms of latency. Interestingly, the RESTlet solution provides a very good opportunity to use visual programming techniques to reduce the IoT application development to a set of drag-and-drop or point-and-click activities.

We do realize that this solution can be optimized further. One possible optimization could be achieved by looking at cross-layer processing activities. This is one of the potential areas of work in the future. In this paper, we stored the RESTlet code in the nodes at compile time which makes it inefficient in case that node is not selected to host that particular RESTlet. A more optimized solution would consist of the dynamic deployment of selected RESTlets at run-time. This is another area for future work. Optimal placement of RESTlet nodes in the network is also another future research topic. From the experiments we conducted, we found out that whenever the RESTlet is closer to the data generating nodes, the RESTlet solution performs better. In the future, we will come up with mathematical models which will lead to optimal placement of the RESTlet nodes.

## Figures and Tables

**Table 1:** Memory Foot Print.

Num. Observers	Binding Sensor				Non-Binding Sensor			
	Text	Data	BSS	Total	Text	Data	BSS	Total
0	47,829	306	5324	53,459	46,453	306	5166	51,925
1	47,829	306	5580	53,715	46,453	306	5384	52,143
2	47,829	306	5836	53,971	46,453	306	5602	52,361
3	47,829	306	6092	54,227	46,453	306	5820	52,581
4	47,829	306	6348	54,483	46,453	306	6058	52,797
